# Dietary vitamin D is a novel modulator of tumor engraftment through regulation of GC protein abundance

**DOI:** 10.21203/rs.3.rs-3911213/v1

**Published:** 2024-02-16

**Authors:** Lo Danahy, Caela Long, Ted J. Hofmann, Zahra Tara, Julian Mark, Jeffrey D. Roizen

**Affiliations:** The Children’s Hospital of Philadelphia; The Children’s Hospital of Philadelphia; The Children’s Hospital of Philadelphia; The Children’s Hospital of Philadelphia; The Children’s Hospital of Philadelphia; The Children’s Hospital of Philadelphia

## Abstract

The vitamin D binding protein, the GC protein, is a multifunctional protein that binds circulating vitamin D and also increases macrophage killing of tumor cells. Injecting exogenous GC protein concurrent with experimental tumor implant decreases tumor engraftment rate. Until now serum abundance of this protein was thought to be controlled by estrogen, glucocorticoids and inflammatory cytokines, but, not by vitamin D itself(1, 2). Nonetheless, increasing dietary vitamin D is thought to increase serum vitamin D, which is 98% bound by the GC protein.

Based on the protection that excess GC protein offers we sought to determine if decreased GC protein abundance might decrease tumor immunity. Relatedly, we theorized, by contrast to the current model, that dietary vitamin D might affect serum abundance of GC protein. If exogenous vitamin D alters available GC levels, then this effect might indicate a novel pathway by which vitamin D enhances immunity. To examine these possibilities, we examined the effect of GC protein absence on tumor persistence or engraftment on two different and common tumor types (prostate cancer and breast cancer). We further examined the relationship between dietary vitamin D and serum GC abundance. We found that absence of GC protein allowed significantly more engraftment of breast tumor cells in female mice and of prostate tumor cells in male mice. Further, we found a U-shaped response of serum GC protein to dietary vitamin D dosage as well as to serum vitamin D, indicating the potential benefit of high exogenous doses to enhance immunity and reduce tumor burden.

## Introduction

Cancer is the second leading cause of death in the developed world and is rapidly moving towards becoming the leading cause of death. Recently developed tumor directed cell-based therapies such as chimeric antigen receptor (CAR) T-cells have enabled effective therapy for previously difficult to treat cancers. Using these kinds of synthetic receptors in other immune cells such as macrophages promises to further increase the types or severity of cancers that we can effectively treat. However, one unexpected challenge with these therapies is that in specific tumor contexts such as solid tumors the overall activity or aggressiveness of the enhanced immune system can be muted(3).

The vitamin D binding protein, the *GC protein*, is a multifunctional protein that binds circulating vitamin D and also increases macrophage killing(4–6). Exogenous GC protein given concurrent with experimental tumor implant decreases tumor engraftment rate. Until now serum abundance of this protein was thought to be controlled by estrogen, glucocorticoids and inflammatory cytokines, but, not by vitamin D itself(1, 2). However, more than 98% of all serum vitamin D is bound by GC protein and increasing dietary vitamin D is thought to increase serum vitamin D.

Based on the protection that excess GC protein offers we sought to determine if decreased GC protein abundance might decrease tumor immunity. If exogenous vitamin D alters available GC levels, then this might indicate a pathway by which vitamin D affects immunity. To examine these possibilities, we measured the effect of GC protein absence on tumor engraftment in mice for two distinct and commonly found tumor types: prostate cancer and breast cancer. We further examined the relationship between dietary vitamin D and serum GC abundance.

## Results

### GC ^−/−^ mice have increased prostate and breast cancer tumor persistence.

**Figure 1:**
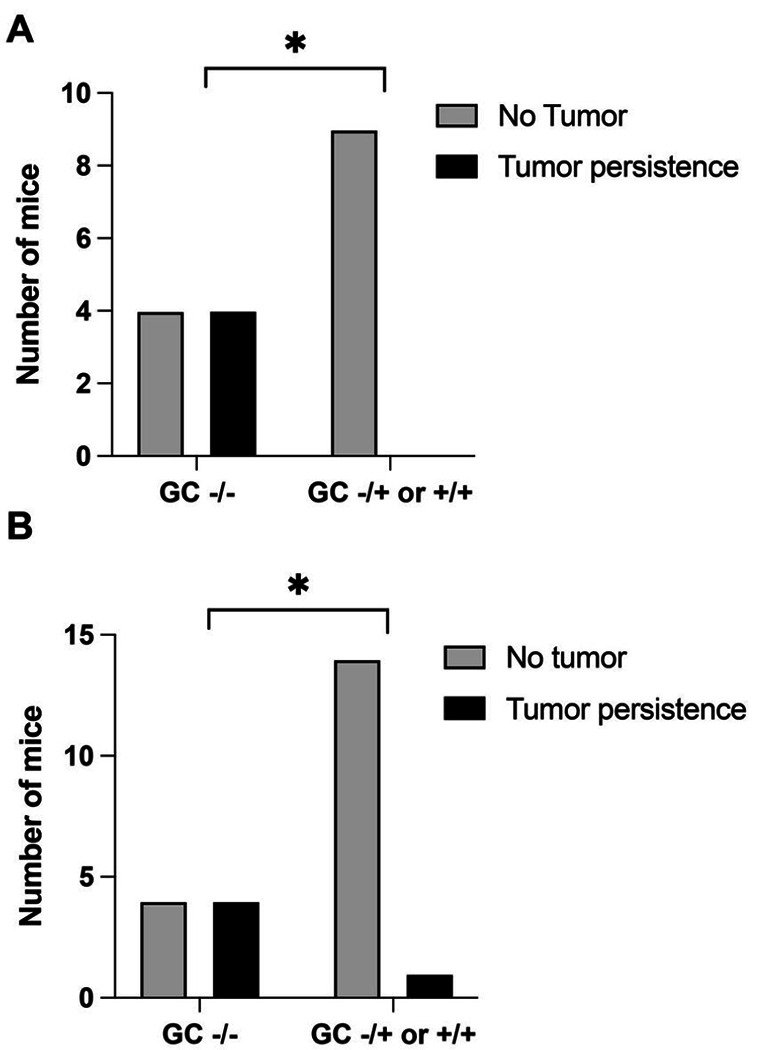
Tumor engraftment is enhanced in the absence of GC protein. Mice were implanted with tumor cells and then sacrificed after 8 weeks. **A:** Breast cancer implants are significantly more likely to engraft in GC−/− mice than in their inbred littermates (* p < 0.05 by Fisher’s Exact test). **B**: Prostate cancer implants are significantly more likely to engraft in GC−/− mice than in their inbred littermates (* p < 0.05 by Fisher’s Exact test).

We obtained GC^+/−^ mice on a C57BL6J (Jackson) background from the International Knockout Consortium (IKC). To examine the effect of knockout of the GC protein on tumor survival we used models for two common tumors: breast cancer(7) and prostate cancer(8, 9). As described previously we implanted tumor plugs of cultured cells into mice with (GC^+/+^, GC^+/−^) and without (GC^−/−^) the GC protein. Female mice without the GC protein had significantly increased breast tumor engraftment relative to mice with the GC protein (Fig. 1A, * p = 0.029 Fisher’s Exact test). Male mice without the GC protein had significantly increased prostate tumor engraftment relative to mice with the GC protein (Fig. 1B, * p = 0.032 Fisher’s Exact test). There were not significant differences between tumor types suggesting that the GC protein is likely to play a similar role in immune attack of diverse tumors.

### Dietary vitamin D determines GC concentration

**Figure 2:**
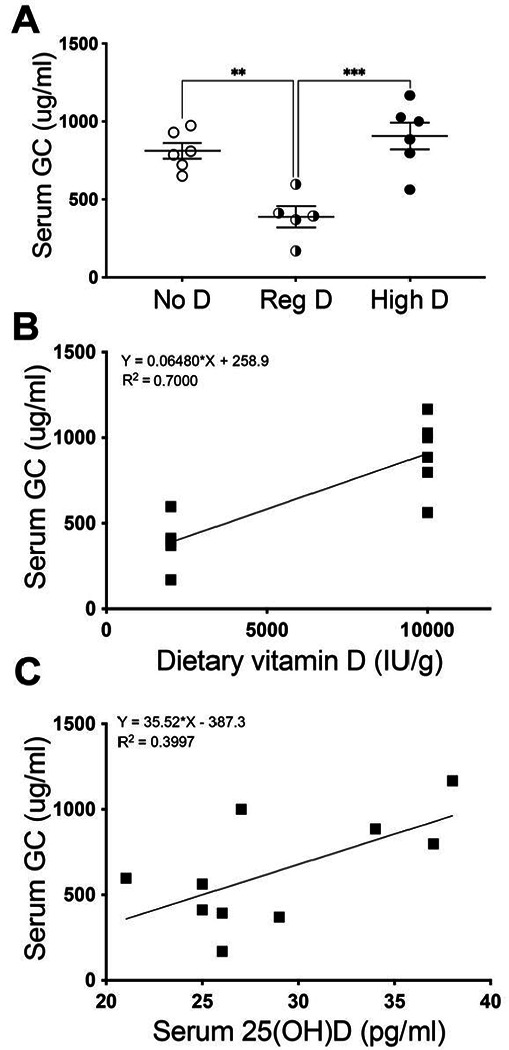
Dietary vitamin D regulates serum GC protein abundance. Mice were put on diets with one of three concentrations of cholecalciferol and GC protein and serum calcidiol were measured at sacrifice after 12 weeks on the diet. **A**: Dietary vitamin D (cholecalciferol) level affects serum GC protein abundance in a U-shaped relationship. **B**: Dietary vitamin D contributes significantly to serum GC protein abundance (R^2^ = 0.7, **p<0.01)). **C**. Serum vitamin D contributes significantly to serum GC protein abundance (R^2^ = 0.39, *p < 0.05).

Serum concentrations of the vitamin D binding protein are thought to be regulated by testosterone, estrogen, glucocorticoids and inflammatory cytokines, but, not by vitamin D itself. Calcidiol (25(OH)D) is the major circulating form of vitamin D. In the absence of significant liver or kidney pathophysiology, the vast majority (> 98%) of circulating calcidiol is bound by the GC protein. Increasing dietary vitamin D or significant skin exposure to appropriate wavelengths of sunlight each raise serum vitamin D. Therefore we wondered if, contrary to the current dominant model, dietary vitamin D might alter serum GC protein abundance. To examine this possibility we measured GC protein concentrations in the serum of mice maintained on the vitamin D receptor knockout rescue diet with three different doses of vitamin D. As we theorized, varying vitamin D affected serum GC protein concentration. There were significant differences in serum GC protein concentration between the three doses (Fig. 2A: ***p < 0.001 by One-way ANOVA, with post-tests adjusted with Sidek’s approximation significant for differences between no-D and reg-D (** p < 0.01and reg-D and high-D (*** p < 0.001). Surprisingly, the relationship between dietary vitamin D and GC protein abundance was a U-shaped curve. GC protein was increased with absence of vitamin D from the diet or at high doses of vitamin D but was decreased at moderate doses of dietary vitamin D (Fig. 2A). We used linear regression to examine the relationship betweef dietary vitamin D dose and GC concentration for diets that contained vitamin D (e.g. Reg D, 2000 IU/kg and High D, 10,000 IU/kg) revealed that dietary vitamin D dose significantly affects GC protein concentration (Fig. 2B, R^2^ = 0.7, **p < 0.01). We also examined the relationship between serum 25(OH)D concentration and GC protein abundance for in mice on diets containing vitamin D. This approach revealed a significant relationship (*p < 0.05) between serum vitamin D concentration and serum GC protein concentration (Fig. 2C).

## Discussion

Here we describe for the first time increased tumor engraftment in multiple tumor types in the context of absence of the GC protein. Previous work described decreased tumor engraftment or increased survival post-tumor implantation due to infusion with GC protein(10–12). Our results add negative effects of low GC protein abundance to the dynamic range of possible effects of this protein. Additionally we identify a novel U-shaped relationship between dietary cholecalciferol and serum protein GC protein abundance as well as a weaker relationship between serum calcidiol and serum protein GC protein abundance. There are several possibilities why this relationship between GC protein and dietary cholecalciferol or serum calcidiol may have previously been missed. First, it may be that researchers assumed a linear relationship between these two variables rather then a U shaped curve. Alternately, it may have been missed because investigators examined the effects of a single dose regimen (e.g. 4000 IU, daily) which for a hypothetical population with variable initial GC protein concentrations may cause some individuals to move to a lower GC-protein concentration and others to move to a higher GC-protein concentration. Finally, it is possible that prior work occurred prior to our understanding of GC-protein allele effects and did not account for the variability due to different GC-protein alleles: we would expect these alleles to each may operate on their own GC-protein U shaped curve. Our work manages to avoid these first two pitfalls by using three doses of vitamin D and avoids this last pitfall by using inbred mice with a limited GC-protein allele repertoire.

Strengths of this work include identifying GC protein effects across cancer types as well as the novelty of our two findings. Weakness of this work include a lack of mechanistic explanations about how dietary vitamin D might alter GC protein abundance.

In other work we had previously identified decreased generation of calcidiol (25-OH-D_3_) from dietary cholecalciferol in the context of obesity(13). This previously identified change in calcidiol generation may result from the inflammatory milieux associated with obesity. The downstream effect of low serum calcidiol concentrations to decrease GC protein may be another way that obesity and other inflammatory diseases lead to worsened cancer outcomes. Alternately, given the role of the liver in converting cholecalciferol to calcidiol and the relative strength of the relationship between dietary vitamin D and GC protein, GC protein abundance may reflect signaling related to conversion of cholcalciferol to calcidiol. Finally, given that GC protein effects occur across cancer types this work illustrates the potential for high vitamin D doses or other methods to increase GC protein such as infusion of GC protein to decrease tumor spread, or to enhance CAR-based approaches to cancer treatment.

## Methods

### Study Approval

All procedures were carried out in accordance with the National Institutes of Health Guidelines on the Care and Use of Animals, are reported in accordance with the ARRIVE guidelines and approved by the Children’s Hospital of Philadelphia Institutional Animal Care and Use Committee (Protocol IAC-17-000988). Male mice were used for prostate cancer cell experiments and female mice were used for breast cancer cell experiments. Mice were humanely euthanized at 8 weeks using isoflurane.

#### Animals: Generation of GC−/− mice

8-week old mice were used in our studies. GC^−/−^ embryos on the inbred C57BL6/J background were obtained from the Regeneron arm of the International Knockout Mouse Consortium (IKMC) (REF). Experiments were performed on GC^−/−^ mice and littermate GC^+/−^, GC^+/+^ controls. Deletion was confirmed and animals were genotyped by PCR for each genotype with the following primers: 5’-tgggattagcgtgtttcaactgagc-3’, 5’-ttttggttttggatgagtctgtggag-3’.

### Breast Cancer cells:

EO771.LMB cells (C57BL/6 breast cancer cells) were generously provided by Dr. Anderson(7). Cells were cultured as previously described(7). At 80% confluence, 20,000 cells were mixed 1 to 1 with 100uL of Matrigel. Syringes were loaded with this mixture and 50000 cells were injected into the left buttock of female mice. At 8 weeks post-injection mice were sacrificed and their buttock sectioned to examine for tumors.

### Prostate Cancer cells:

TRAMP-C1 cells were obtained from (ATCC) and cultured as described previously. At 80% confluence 100,000 TRAMP-C1 cells were diluted into 100uL of serum-free media. These cells were mixed 1 to 1 with 100uL of Matrigel. Syringes were loaded with this mixture and 100000 cells were injected into the left buttock of male mice. At 8 weeks post-injection mice were sacrificed and their buttock sectioned to examine for tumors.

### GC protein ELISA

At 12 weeks of age, C57BL/6 wild type mice were moved from a defined CHOW diet to a vitamin D receptor knockout rescue diet with one group of mice on each of three concentrations of cholecalciferol in the diet (0 IU/g, 2000 IU/g and 10,000 IU/g). Mice were maintained on these diets for 12 weeks and then euthanized with serum harvested by right ventricle puncture.

GC protein was assayed using a quantitative elisa (LSBio). Assay was performed on each sample in technical triplicate.

### Vitamin D measurements

At 12 weeks of age, C57BL/6 wild type mice were moved from a defined CHOW diet to a vitamin D receptor knockout rescue diet with one group of mice on each of three concentrations of cholecalciferol in the diet (0 IU/g, 2000 IU/g and 10,000 IU/g). Mice were maintained on these diets for 12 weeks and then euthanized with serum harvested by right ventricle puncture.

Serum calcidiol was assayed by LC-MS-MS as described previously(14).

### Statistics

All values are presented as Mean ± SEM and represent data from a minimum of two repeated experiments. Means were compared using a one-way ANOVA with SIDEK’s correction for post-tests. Proportions were compared using Chi-squared analysis. Fisher’s Exact test

## Data Availability

The datasets generated during and/or analyzed during the current study are available from the corresponding author on reasonable request.
